# Burnout, Work Addiction and Stress-Related Growth Among Emergency Physicians and Residents: A Comparative Study

**DOI:** 10.3390/bs15060730

**Published:** 2025-05-24

**Authors:** Raluca Mihaela Tat, Adela Golea, Gabriela Vancu, Mihai-Bujor Grecu, Monica Puticiu, Andrei Hermenean, Luciana Teodora Rotaru, Mihai Alexandru Butoi, Mihaela Corlade-Andrei, Diana Cimpoesu

**Affiliations:** 1 Department 6 Surgery, Emergency Medicine Discipline, “Iuliu-Hațieganu” University of Medicine and Pharmacy, 400012 Cluj-Napoca, Romania; raluca.tat@umfcluj.ro (R.M.T.); adela.golea@umfcluj.ro (A.G.); 2 Department of Psychology, Faculty of Educational Sciences, Psychology and Social Assistance, University Aurel Vlaicu Arad, Elena Drăgoi Street, No. 2, 310032 Arad, Romania; gabriela.vancu@uav.ro; 3 UPU-SMURD “Pius Brînzeu” Emergency Hospital, 300723 Timisoara, Romania; mihai.grecu@urgentatm.ro; 4 Department of Emergency, Faculty of Medicine, Vasile Goldiș Western University of Arad, 310325 Arad, Romania; 5 Depression Ward 8, General Psychiatric Department, Aalborg University Hospital South, 9000 Aalborg, Denmark; 6 Emergency Medicine and First Aid Department, Faculty of Medicine, University of Medicine and Pharmacy, 200349 Craiova, Romania; luciana.rotaru@umfcv.ro (L.T.R.); mihai.butoi@rmu.smurd.ro (M.A.B.); 7 Surgery Department—Emergency Medicine Discipline, University of Medicine and Pharmacy “Grigore T. Popa”, 700115 Iasi, Romania; mihaela.corlade2@umfiasi.ro (M.C.-A.); carmen.cimpoesu@umfiasi.ro (D.C.); 8 Emergency “St. Spiridon” Hospital, 700111 Iasi, Romania

**Keywords:** emergency medicine, physicians, residents, burnout, work addiction, stress-related growth

## Abstract

The field of emergency medicine (EM) is a high-stress medical specialty. We aim to comparatively investigate burnout, work addiction, and stress-related growth between EM physicians and EM residents. Our sample consists of 117 EM professionals, 41 physicians and 76 residents, from 5 out of the 12 EM county departments in Romania that run residency programs. Methods: An online survey was sent to 461 EM professionals (170 physicians and 291 residents), with a response rate of 25.4%. The survey comprised two sections: the first focused on sociodemographic and professional data, with the second consisting of six validated assessment instruments: the Oldenburg Burnout Inventory, Dutch Work Addiction Scale—short version, Stress-Related Growth Scale, Responsive Distress Scale, Self-Discipline Scale, and Zuckerman–Kuhlman Personality Questionnaire. Both EM physicians and residents reported moderate to high levels of burnout, disengagement, and exhaustion, but there was no significant difference between them. However, physicians exhibited significantly higher levels of work addiction, excessive work, compulsive work, and stress-related growth. No significant differences were found in compulsive work behaviors. Conclusions: Burnout levels are comparable between EM physicians and residents. However, physicians demonstrate higher work addiction but also higher stress-related growth. Personality variables and sleep duration appear to be more influential in predicting burnout than in work addiction or stress-related growth.

## 1. Introduction

Burnout is commonly defined as a three-dimensional syndrome, referring to emotional exhaustion, disengagement from work, and a sense of ineffectiveness (Kase & Doolittle, 2023). It was first used by Freudenberg to describe a state of mental and physical exhaustion caused by high levels of stress and chronic fatigue in the case of a caring professional (Rajvinder, 2018). Maslach developed a coherent theory of burnout as a three-dimensional construct: emotional exhaustion, depersonalization, and low personal achievement (Demerouti et al., 2001).

A broader model of burnout, proposed by Demerouti et al. (2001), known as the job demands and resources model (JR-D model), focuses only on two core dimensions: exhaustion and disengagement, arguing that ineffectiveness or low accomplishment is an individual outcome of burnout. Excessive job demands will lead to exhaustion, while lack of resources, work-related or individual, will lead to disengagement. Negative outcomes of burnout affect healthcare providers (job dissatisfaction, physical and mental health issues), patients (medical errors, decreased care for patients), and medical systems (absenteeism, high turnover rates, and the inefficient use of resources) (West et al., 2018; Panari et al., 2019). In our study we opted for the JR-D model.

A substantial body of research highlights the high prevalence of burnout among emergency medicine (EM) professionals. For example, Boutou et al. (2019) reported rates between 25% and 77.8%, while Verougstraete and Hachimi Idrissi (2020) reported rates between 25.4% and 71.4% among EM physicians, and ones between 55.6% and 77.9% among EM residents. Somville et al. (2021) noted that burnout rates among EM physicians vary between 43% and 54%, with evidence of a rising trend over the past decade, while Zhang et al. (2020) reported an average burnout rate of approximately 40%. Regarding the EM residents, Vanyo et al. (2020) found that 36.4% met the criteria for burnout, while Lu et al. (2023) reported a burnout rate of 31.7%. Other studies indicates moderate to high levels of burnout among both EM physicians and residents (Kuhn et al., 2009; Arora et al., 2013; Takayesu et al., 2014; Wilson et al., 2017; Baier et al., 2018; Williamson et al., 2018; Lin et al., 2019; Liu et al., 2020; Chang et al., 2022; Sakamoto et al., 2022; Alanazy & Alruwaili, 2023; Al-Salamah et al., 2024).

In Romania, a nationwide study conducted in 2010, involving 4693 EM health workers, representing 29.9% of national emergency personnel, revealed moderate to high rates of burnout (Popa et al., 2010). More recent data from Puticiu et al. (2024) indicate that among EM physicians, 43.8% experience moderate levels of burnout, while 19.5% report high levels.

Work addiction is characterized by an excessive involvement in work that goes well beyond what is required to be efficient and satisfied, leading to physical and mental health problems (Griffiths et al., 2018). The term was derived from workaholism, first used by Oates. However, many scholars argue that the two are related but distinct constructs, with partial conceptual overlap (Schaufeli et al., 2009b; Griffiths, 2023). Currently, work addiction is widely recognized as a behavior addiction, arising from a combination of individual and work-related factors. Emerging evidence suggests a connection between burnout and work addiction, with work addiction mediating the relationship between perfectionism and burnout (Taris et al., 2010). Among physicians, high levels of work addiction have been associated with reduced quality of life and decreased work efficiency (Azevedo & Mathias, 2017).

Schaufeli et al. (2009a) showed that work addiction is prevalent among medical residents and is associated with several occupational stressors including burnout, high workload, work–home conflict, mental and physical demands, low social support from peers, and the need to make critical decisions in high-stress environments. Rezvani et al. (2014) found that 13% of 444 French university hospital physicians exhibited high levels of work addiction, while 35% showed mild addiction. Similarly, in a study involving 1108 Brazilian physicians, it was found that 44.9% displayed moderate to strong levels of work addiction (Azevedo & Mathias, 2017). Merchaoui et al. (2021) further highlighted the variability in prevalence, noting that rates of work addiction among physicians vary widely from 8.3% to 30%, depending on the population and measurement tools used.

In a sample of 266 Romanian prehospital EM personnel (physicians, nurses, and paramedics), 35% met the threshold for work addiction, with the highest prevalence observed among physicians, at 63.4% (Puticiu et al., 2024).

While stress is often associated with negative outcomes—such as physical, psychological, and social impairments—it can also lead to positive psychological changes. Several studies shown that certain amounts of stress can lead to positive outcomes and improvements in coping strategies (Park, 1998; Tedeschi & Calhoun, 2004; Bi et al., 2006), even in highly stressful situations, such as during the COVID-19 pandemic (Brooks et al., 2020; Ushimoto et al., 2023; Manole & Curșeu, 2024), an effect known as stress-related or post-traumatic growth.

The resilience cycle proposes that individuals confronted with a stressful event may experience one of three outcomes: growth, recovery, and impairment. The first element is the initial state/baseline level which is influenced by biological, neuropsychological, and environmental factors. The second element, the adjustment process, depends on the initial state, but also on the severity of a stressful event: low levels of stress lead to recovery in the absence of growth, moderate/tolerable levels of stress lead to growth, and severe stress leads to impairment (Ord et al., 2020). During the adjustment process, growth is modulated by deliberative rumination, cognitive reappraisal, psychological flexibility, emotion regulation, locus of control, problem-solving skills, self-reflection, and finding meaning. A resilient individual is able to maintain adaptive physiological and psychological functioning in the face of stress, thereby reducing the risk of developing physical or mental health problems (Bi et al., 2006; Ord et al., 2020).

Stress-related growth is influenced by several factors, including gender, age, ethnicity, social support, coping mechanisms, levels of stress, cognitive abilities, and personality traits (Park, 1998; Bi et al., 2006; Butoi et al., 2025). Puticiu et al. (2024) found that 61.2% of 266 Romanian EM personnel reported relevant levels of stress-related growth, with the highest rate being for EM physicians, at 80.5%.

Personality factors play an important role in explaining levels of burnout, work addiction, and stress-related growth. Bianchi (2018) found that neuroticism explained 53.46% of burnout variance. A systematic review of 83 papers (Angelini, 2023) showed that higher levels of neuroticism and lower levels of agreeableness, conscientiousness, extraversion, and openness were associated with higher levels of burnout. Jackson et al. (2016) and Kun et al. (2020) found high neuroticism, extraversion, and conscientiousness to be risk factors for work addiction. Positive affectivity, spirituality/religiousness, social support, problem-solving skills, coping strategies, meaningful reinterpretation, acceptance, and openness were positively associated with stress adaptation and growth (Park et al., 1996; Lecic-Tosevski et al., 2011).

**Figure 1:**
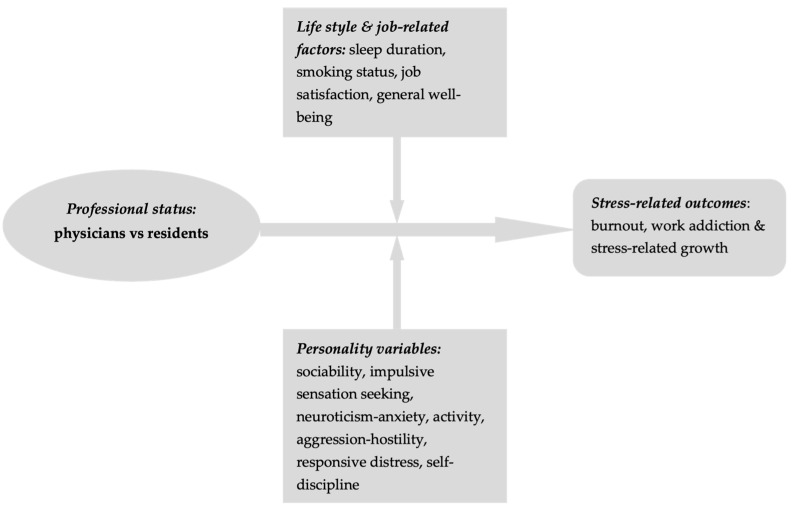
Methodological framework.

The aim of this study is to comparatively investigate burnout, work addiction, and stress-related growth among emergency medicine (EM) physicians and residents in Romania, while also considering the impact of age, sleep duration, job satisfaction, general well-being, and personality traits (Figure 1).

We hypothesize that there are significant differences between EM physicians and EM residents regarding burnout, work addiction, and stress-related growth levels. To our knowledge, this is the first study in Romania to simultaneously assess three key stress-related outcomes among EM professionals at different stages of their medical careers.

## 2. Materials and Methods

### 2.1. Participants and Procedure

This is a comparative cross-sectional study. We addressed 5 out of the 12 EM county departments in Romania that run residency programs, totaling 461 EM professionals, 170 physicians (36.9%) and 291 residents (63.1%). Participants were invited to fill out an online survey consisting of two sections: the first section gathered demographic and professional data (age, gender, years of practice/residency, smoking status, sleep duration outside working shifts, general well-being, and work-satisfaction), while the second section included six assessment tools (the Oldenburg Burnout Inventory—OLBI; Dutch Work Addiction Scale—short version—DUWAS-10; Stress-Related Growth Scale—SRGS; Responsive Distress Scale—RDS; Self-Discipline Scale—SDS; and the Zuckerman–Kuhlman Personality Questionnaire—ZKPQ). In total, 117 individuals (41 physicians and 76 residents) filled out the online survey, reflecting a response rate of 25.4% (24.1% for physicians, 26.1% for residents).

### 2.2. Measures

The Oldenburg Burnout Inventory (OLBI) is a 16-item self-reported questionnaire that assesses burnout on two dimensions: disengagement (8 items) and exhaustion (8 items). Items are scored on a 4-point Likert scale, from 1—strongly disagree to 4—strongly agree. The total score can range from 16 to 64, while each subscale score ranges from 8 to 32. Scores above the 75th percentile are considered indicative of clinically relevant burnout levels (Demerouti et al., 2001; Puticiu et al., 2024).

The Duch Work Addiction Scale—short version (DUWAS-10) is a 10-item scale, designed to measure work addiction across two dimensions: working excessively and working compulsively, each measured with 5 items. Items are scored on a 4-point Likert scale, ranging from 1—never to 4—always. Dimensions and total scores are calculated by averaging the item responses, resulting in values ranging from 1 to 4. Scores above the 75th percentile are considered indicative of significant levels of work addiction (Schaufeli et al., 2009c; Del Líbano et al., 2010).

The Stress-Related Growth Scale (SRGS) was developed by Park et al. (1996) as a 15-item scale for assessing stress-related growth. Items are scored on a 3-point Likert scale, ranging from 0—disagree to 2—strongly agree, yielding a total score from 0 to 30. A cut-off score of 28 is used to indicate a high level of stress-related growth. The Romanian version is part of the Clinical Assessment System developed by RTS Romanian Psychological Testing Services.

The Responsive Distress Scale (RDS) was developed by Barchard (2001) as a 10-item tool for assessing responsive distress, which refers to the tendency of individuals to experience negative emotions when faced with others’ distress. Items are dichotomous, scored with a yes or no, resulting in total scores ranging from 0 to 10 (Iliescu et al., 2015).

The Self-Discipline Scale (SDS) is a 10-item scale originally developed by Harrison Gough for the California Personality Inventory, and now a part of the International Personality Item Pool (IPIP). It is designed to assess an individual’s ability to exercise self-control and regulate behavior in accordance with rules and procedures, rather than emotional impulses (Iliescu et al., 2015). Items are answered in a dichotomous format (*yes* or *no*), yielding a total score ranging from 0 to 10.

The Zuckerman–Kuhlman Personality Questionnaire (ZKPQ) is designed to assess human personality in five broad domains—sociability (Sy), impulsive sensation seeking (ISS), activity (Act), neuroticism–anxiety (N-Anx), and aggression–hostility (Agg-H)—as theorized by Marvin Zuckerman’s Alternative Five Factor Model (Zuckerman et al., 1993; Zuckerman, 2002). The questionnaire consists of 99 items (true versus false answers). The Romanian version has been adapted by (Miclea et al., 2009).

The Romanian versions of the OLBI, DUWAS-10, RDS, and SDS are available at https://researchcentral.ro. Work satisfaction and general well-being were self-rated on a 10-point Likert scale, where 1—extremely low and 10—extremely high.

### 2.3. Statistical Procedures

Data collected via the online survey were systematized and analyzed using IBM SPSS Statistics 20 software. The statistical procedures used were Fisher’s exact test, independent samples *t*-test, Mann–Whitney U test, one-sample Kolmogorov–Smirnov test, Spearman’s rho, and generalized linear models when controlling for covariates due to the fact that outcome variables did not follow the normal distribution law (Miles, 2005). *p*-value significance was set at 0.05.

## 3. Results

Table 1 presents descriptive data (percentages, mean, and standard deviation) for physicians and residents regarding the sociodemographic and lifestyle variables. Among physicians, the years of professional practice ranged from 7 to 34, with an average of 17.59 ± 7.86, while for residents, the years of residency ranged from 0 to 5, with an average of 2.89 ± 1.53. We considered age and practice/residency years as a function of the physician/resident status, and not as covariates.

Age and average sleep duration differed significantly between groups, with higher values among physicians. No significant differences were found regarding gender distribution, smoking status, work satisfaction, or general well-being. Distributions for work-satisfaction and general well-being did not follow the normal distribution law, as revealed by the results of the Kolmogorov–Smirnov test; thus, a non-parametrical test was used. The GLZ results show no significant difference in sleep duration when controlling for personality variables. No significant differences were found in general well-being and work satisfaction when controlling for sleep duration (Table 1).

Table 2 shows that age strongly correlates with personality factors and traits, except for self-discipline, for all participants. But no such correlations were found for physicians as a distinct group. For residents, responsive distress and self-discipline did not correlate with age.

Considering all of the participants, the average sleep duration outside working shifts strongly correlated with all personality variables, but only for neuroticism–anxiety in the case of physicians. Work satisfaction and general well-being showed no significant correlations with personality variables for all of the participants; however, for physicians, work satisfaction negatively correlated with aggression–hostility (*p* = 0.012) and responsive distress (*p* = 0.031).

Table 3 presents the descriptive data (mean and standard deviation) and group comparison between physicians and residents regarding personality variables. Physicians show higher mean values for sociability, activity, and self-discipline, while residents for impulsive sensation seeking, neuroticism–anxiety, aggression–hostility, and responsive distress. Significant differences were found for sociability, impulsive sensation seeking, neuroticism–anxiety, and responsive distress, even when controlling for sleep duration.

Table 4 presents descriptive data (mean and standard deviation) for stress-related variables among physicians and residents. Physicians show higher mean values for all variables. When adjusting for sleep, work satisfaction, general well-being, and personality variables, significant differences were found for work addiction as well as excessive and compulsive work. The estimated marginal means were as follows: (1) work addiction: physician—2.66, residents—2.26; (2) working excessively: physicians—2.34, residents—1.89; and (3) working compulsively: physicians—2.98, residents—2.62.

Relevant levels of burnout (scores above the 75th percentile, >58) were observed in 14 physicians (34.1%) and 16 residents (21.0%). For the disengagement and exhaustion subscales, scores ≥ 29 were considered indicative of elevated risk. These thresholds were met by 21 physicians (51.2%) and 24 residents (31.6%) for disengagement, and by 22 physicians (53.6%) and 28 residents (36.8%) for exhaustion.

For DUWAS-10, scores above the 75th percentile were considered indicative of relevant levels of work addiction and its subcomponents. The cut-off scores were 2.8 for work addiction, 2.5 for working excessively, and 3.4 for working compulsively. These thresholds were met by 29 physicians (70.7%) and 18 residents (23.7%) for work addiction, 29 physicians (70.7%) and none of the residents for working excessively, and 24 physicians (58.5%) and 18 residents (23.7%) for working compulsively.

The cut-off score for relevant stress-related growth was 28 (Park et al., 1996), and was met by 34 physicians (82.9%) and 9 residents (11.8%).

Considering all of the participants, age positively correlated (Spearman’s rho) with work addiction, working excessively and compulsively, and stress-related growth (*p* < 0.001). When analyzed separately, age correlated only with stress-related growth (*p* < 0.001) for both groups.

Average sleep duration positively correlated with disengagement (*p* = 0.002), work addiction (*p* = 0.001), working excessively (*p* < 0.001), working compulsively (*p* = 0.004), and stress-related growth (*p* < 0.001) in general. However, for physicians, correlation was significant and positive for burnout (*p* = 0.001), disengagement (*p* = 0.001) and exhaustion (*p* = 0.002), while for residents, there were positive correlations for work addiction (*p* = 0.032), working compulsively (*p* = 0.018), and stress-related growth (*p* < 0.001).

As shown in Table 5, significant positive correlations were found for burnout and activity, disengagement, and activity, as well as self-discipline, work addiction, and activity, as well as self-discipline, working excessively and activity, sociability, and self-discipline, working compulsively and activity, as well as self-discipline, and stress-related growth and activity, as well as sociability (Table 4).

Negative correlations were found for burnout and impulsive sensation seeking, as well as responsive distress, disengagement impulsive sensation seeking, neuroticism–anxiety and responsive distress, exhaustion and responsive distress, work addiction and impulsive sensation seeking, neuroticism–anxiety, as well as responsive distress, working excessively and impulsive sensation seeking, neuroticism–anxiety, aggression–hostility and responsive distress, working compulsively and impulsive sensation seeking, neuroticism–anxiety and responsive distress, and stress-related growth with impulsive sensation seeking, neuroticism–anxiety, aggression–hostility, and responsive distress (Table 5).

## 4. Discussion

We investigated a total of 117 EM medical professionals—comprising 41 physicians and 76 residents—from 5 out of the 12 EM county departments in Romania that run residency programs. The aim was to identify relevant differences between the two groups in relation to three key stress-related outcomes commonly associated with high-stress professions such as emergency medicine: two negative outcomes (burnout and work addiction) and one positive outcome (stress-related growth).

As expected, physicians were significantly older than residents. Additionally, they reported a longer sleep duration outside of work shifts—approximately 8 h, compared to 7 h for residents. No significant differences were observed in terms of smoking status. Both groups scored relatively high on work satisfaction and general well-being. However, it is important to note that these variables were self-assessed on a single item, which may limit their interpretive value.

Personality-wise, physicians were more sociable and emotionally stable, less impulsive, and showed lower responsive distress when faced with stressful situations. There were no differences regarding activity, aggression–hostility, or self-discipline. Lower levels of neuroticism and anxiety for physicians might explain lower responsive distress levels, as the latter seem to be an outcome of neuroticism (Butoi et al., 2025). However, lower levels of responsive distress could indicate that physicians are better accustomed to highly stressful situations specific to EM activity compared to residents, thus showing that exposure can lead to improved coping strategies and lower emotional reactivity.

In both groups, scores for sociability, activity, and impulsive sensation seeking were in the moderate to high range, while neuroticism–anxiety and aggression–hostility were generally low to moderate. These findings suggest that emergency medical professionals tend to be emotionally stable, exhibit good self-control, and are characterized by high self-discipline, sociability, activity, and a tendency to seek and embrace challenging situations. However, residents appear to manage such situations less effectively than physicians, as indicated by their higher levels of responsive distress, suggesting a greater emotional impact when exposed to others’ suffering or crisis, at least at the beginning of their medical career.

Personality factors and traits are strongly correlated with outcome variables. Burnout was associated with low impulsive sensation seeking and responsive distress, and high activity. Disengagement was linked with low impulsive sensation seeking, neuroticism, and responsive distress, as well as a high activity and self-discipline. Exhaustion was primarily associated with low responsive distress.

Work addiction was associated with low levels of impulsive sensation seeking, neuroticism, and responsive distress, and high activity, sociability, and self-discipline. The working excessively dimension correlated with low impulsive sensation seeking, neuroticism, aggression–hostility, and responsive distress, and high activity, sociability, and self-discipline. Similarly, working compulsively was linked to low impulsive sensation seeking, neuroticism and responsive distress, alongside high activity and self-discipline.

Stress-related growth was associated with low levels of impulsive sensation seeking, neuroticism, aggression–hostility, and responsive distress, as well as high levels of activity and sociability. These findings are consistent with previous research showing that personality variables significantly influence burnout (McManus et al., 2004; Alarcon et al., 2009; Swider & Zimmerman, 2010; Bianchi, 2018; Kyron et al., 2021; Angelini, 2023; Butoi et al., 2025), work addiction (Burke et al., 2006; Jackson et al., 2016; Griffiths et al., 2018; Kun et al., 2020; Kızıloğlu et al., 2022), and stress-related growth (Park, 1998; Shakespeare-Finch et al., 2005; Butoi et al., 2025). In contrast to most studies that identify high neuroticism as a predictor of these outcomes, our findings suggest that lower levels of neuroticism are associated with both negative (burnout, work addiction) and positive (stress-related growth) stress-related outcomes in our sample.

However, this effect may be explained by the fact that, in our sample, both physicians and residents scored significantly lower on neuroticism compared to the general Romanian population. This suggests that, within this specific professional group, neuroticism may not function as the primary personality mechanism underlying stress-related outcomes, as previously proposed by Butoi et al. (2025). Nonetheless, further research is needed to clarify this finding and to determine whether it reflects a broader trend among emergency medical professionals or a sampling-specific characteristic.

After controlling for sleep duration, work satisfaction, general well-being, and personality variables, no significant differences were found between physicians and residents in terms of burnout or its two components—disengagement and exhaustion. Both groups reported moderate to high levels of burnout, consistent with findings from previous studies (Takayesu et al., 2014; Boutou et al., 2019; Lin et al., 2019; Vanyo et al., 2020; Verougstraete & Hachimi Idrissi, 2020; Zhang et al., 2020; Lu et al., 2023). These results suggest that factors such as age and personality may have a greater impact on burnout levels than professional seniority or clinical experience.

Work addiction and its subdimension working excessively, but not working compulsively, were significantly higher in physicians, even after controlling for sleep duration, work satisfaction, general well-being, and personality variables.

This suggests that greater clinical exposure and increased responsibility in the medical decision-making process may contribute to more excessive engagement in work-related activities, whereas factors such as age, sleep, and personality appear to play a less prominent role (Butoi et al., 2025). However, the higher levels of work satisfaction and general well-being reported by both physicians and residents may indicate that increased work involvement among physicians is not necessarily associated with negative outcomes, supporting prior findings that differentiate between productive work engagement and harmful work addiction (Schaufeli et al., 2009a; Griffiths, 2023).

Physicians showed significantly higher levels of stress-related growth compared to residents, even after controlling for sleep, work satisfaction, general well-being, and personality. In our sample, 82.9% of physicians showed relevant levels of stress-related growth in comparison with only 11.8% for residents. Although personality traits were shown to be a relevant factor for stress-related growth (Park, 1998; Shakespeare-Finch et al., 2005; Butoi et al., 2025), it seems that when comparing physicians with residents, longer exposure to stressful events, if they fall in that area of manageable stress levels (Ord et al., 2020), play a leading role in helping medical professionals grow and improve their coping strategies.

These results offer partial support for our hypothesis, indicating that, although there were no differences between physicians and residents regarding burnout levels, physicians do show higher levels of work addiction and stress-related growth. Addtionally, our results indicate that while personality is a contributing factor, mainly for burnout, professional experience and exposure to clinical stressors may have a more substantial impact on work addiction and the development of stress-related growth. However, the inclusion of less than half of the total EM departments who run residency programs (5 out of 12) and the relatively low response rates indicate that the generalization of results must be performed with high precautions.

As shown in our findings, negative stress-related outcomes, such as burnout and work addiction, are present at moderate to high levels in both EM physicians and residents, underscoring the urgent need for targeted interventions to support the well-being of these essential healthcare professionals. Recent studies highlight effective strategies in this regard. For instance, Liu et al. (2024) demonstrated that structured wellness programs can significantly reduce burnout levels among EM professionals. Similarly, Egan et al. (2022) found that retreat-based interventions, designed to foster transformative and reflective experiences, are effective in reducing stress during medical residency. Furthermore, there is a clear need for interventions specifically aimed at enhancing residents’ coping mechanisms, particularly through improved emotional regulation and greater engagement in their professional roles.

Further studies, involving larger samples and conducted over a longer time span, are needed to better understand how burnout rates evolve during an EM physician’s career, from residency to experienced practice. Such studies would also be valuable in identifying the factors that contribute to or hinder stress-related growth, with the aim of developing strategies that help EM professionals optimize their adaptive responses to stress and enhance resilience in the face of the demanding conditions inherent to their field.

## 5. Conclusions

Our study shows that burnout levels do not differ significantly between EM physicians and residents, both groups reporting moderate to high levels. However, physicians appear to be more deeply involved in their work, in some cases reaching levels indicative of work addiction, in both ways—excessive work and compulsive engagement in work activities—whereas residents maintain a more balanced work–life relation. Despite this, physicians exhibit higher levels of stress-related growth, suggesting that they may have developed more effective coping strategies for managing the intense and routine stressors inherent in emergency medicine. This could help explain why burnout levels remain comparable between the two groups, even though physicians demonstrate a higher degree of work involvement.

## Figures and Tables

**Table 1:** Comparative data on demographic and professional variables.

Variable	Physicians (N = 41)	Residents (N = 76)	Unadjusted Model *p*-Value	GLZ *p*-Value
Gender	Men—12 (29.3%) Women—29 (70.7%)	Men—22 (28.9%) Women—54 (71.1%)	1 ^1^	-
Age (years)	44.61 ± 8.10 Range: 31–57	30.78 ± 5.79 Range: 25–50	<0.001 ^2^	-
Smoking status	Smokers—26.8%	Smokers—44.7%	0.074 ^1^	-
Sleep average outside working shifts (h)	7.95 ± 1.22 Range: 4–10	7.00 ± 1.07 Range: 5–9	<0.001 ^2^	0.083 ^4^
Work satisfaction	8.59 ± 0.92 Range: 5–10	8.79 ± 1.07 Range: 6–10	0.133 ^3^	0.062 ^5^
General well-being	8.37 ± 1.36 Range: 4–10	8.55 ± 1.43 Range: 5–10	0.330 ^3^	0.746 ^5^

^1^ Fisher’s exact test. ^2^ Independent samples *t*-test. ^3^ Mann–Whitney U test. ^4^ Controlling for personality factors. ^5^ Controlling for sleep duration.

**Table 2:** Correlations between age, work satisfaction, general well-being, and personality variables.

Variable		Personality Factors and Traits						
		ISS	N-Anx	Agg-H	Act	Sy	RDS	SDS
Age	rho *p*	−0.507 <0.001	−0.561 <0.001	−0.444 <0.001	0.360 <0.001	0.446 <0.001	−0.586 <0.001	0.043 0.643
Sleep duration outside working shifts	rho *p*	−0.459 <0.001	−0.468 <0.001	−0.415 <0.001	0.386 <0.001	0.456 <0.001	−0.289 0.002	0.241 0.009
Work satisfaction	rho *p*	−0.139 0.135	−0.162 0.080	−0.153 0.099	0.138 0.139	0.145 0.119	0.002 0.981	0.106 0.254
General well-being	rho *p*	0.135 0.146	0.107 0.251	0.097 0.299	−0.029 0.758	−0.027 0.776	0.088 0.344	0.156 0.092

**Table 3:** Comparison between physicians and residents for personality variables.

Variable	Physicians (N = 41)	Residents (N = 76)	Unadjusted Model ^1^ *p*-Value	GLZ ^2^ *p*-Value
Sociability	13.83 ± 2.57 Range: 3–17	10.25 ± 4.58 Range: 2–17	<0.001	0.002
Impulsive sensation seeking	8.41 ± 2.01 Range: 5–16	11.59 ± 4.29 Range: 5–18	0.001	0.004
Activity	11.39 ± 3.69 Range: 2–16	9.59 ± 4.67 Range: 2–16	0.178	0.528
Neuroticism–anxiety	2.95 ± 1.77 Range: 1–8	5.83 ± 3.74 Range: 1–11	0.001	0.002
Aggression–hostility	1.85 ± 1.29 Range: 1–8	3.16 ± 2.66 Range: 1–9	0.180	0.159
Responsive distress	4.17 ± 1.84 Range: 3–7	6.87 ± 0.68 Range: 3–7	<0.001	<0.001
Self-discipline	9.41 ± 0.95 Range: 6–10	9.26 ± 1.24 Range: 3–10	0.758	0.743

^1^ Mann–Whitney U test. ^2^ Generalized linear model (GLZ), when controlling for sleep duration.

**Table 4:** Comparison between physicians and residents for stress-related variables.

Variable	Physicians (N = 41)	Residents (N = 76)	Unadjusted Model *p*-Value	GLZ *p*-Value ^3^
Burnout	51.61 ± 10.41 Range: 19–61	47.29 ± 11.69 Range: 19–64	0.032 ^1^	0.280
Disengagement	26.95 ± 4.96 Range: 11–32	24.61 ± 5.03 Range: 9–32	0.017 ^2^	0.269
Exhaustion	24.66 ± 6.07 Range: 8–32	22.68 ± 7.25 Range: 8–32	0.279 ^1^	0.337
Work addiction	2.93 ± 0.69 Range: 1.0–3.7	2.12 ± 0.41 Range: 1.6–2.8	<0.001 ^1^	0.006
Working excessively	2.64 ± 0.70 Range: 1.0–3.4	1.73 ± 0.32 Range: 1.0–2.2	<0.001 ^1^	0.001
Working compulsively	3.21 ± 0.80 Range: 1.0–4.0	2.50 ± 0.52 Range: 1.6–3.4	<0.001 ^1^	0.050
Stress-related growth	28.76 ± 2.65 Range: 20–30	21.72 ± 4.04 Range: 14–30	<0.001 ^1^	<0.001

^1^ Mann–Whitney U test. ^2^ Independent samples *t*-test. ^3^ Generalized linear model (GLZ) when controlling for all sleep, work satisfaction, general well-being, and personality variables.

**Table 5:** Correlations between outcome variables and personality variables (Spearman’s rho) for the general sample (N = 117).

Variable		Personality Factors and Traits						
		ISS	N-Anx	Agg-H	Act	Sy	RDS	SDS
Burnout	rho *p*	−0.249 0.007	−0.166 0.074	−0.128 0.169	0.209 0.023	0.069 0.459	−0.271 0.003	0.156 0.093
Disengagement	rho *p*	−0.320 <0.001	−0.261 0.005	−0.162 0.081	0.252 0.006	0.130 0.162	−0.331 <0.001	0.203 0.028
Exhaustion	rho *p*	−0.170 0.066	−0.105 0.258	−0.121 0.193	0.136 0.143	0.050 0.590	−0.189 0.041	0.146 0.116
Work addiction	rho *p*	−0.351 <0.001	−0.282 0.002	−0.178 0.054	0.236 0.010	0.197 0.033	−0.595 <0.001	0.190 0.040
Working excessively	rho *p*	−0.366 <0.001	−0.322 <0.001	−0.191 00.40	0.226 0.014	0.221 0.017	−0.618 <0.001	0.190 0.040
Working compulsively	rho *p*	−0.325 <0.001	−0.212 0.022	−0.140 0.133	0.219 0.017	0.174 0.061	−0.502 <0.001	0.189 0.041
Stress-related growth	rho *p*	−0.394 <0.001	−0.474 <0.001	−0.355 <0.001	0.298 0.001	0.342 <0.001	−0.602 <0.001	0.062 0.505
ISS	Impulsive sensation seeking							
N-Anx	Neuroticism–anxiety							
Agg-H	Aggression–hostility							
Act	Activity							
Sy	Sociability							

RDS: Responsive Distress Scale; SDS: Self-Discipline Scale.

## Data Availability

The data presented in the study are available upon request of the corresponding author.
